# One new species and one new record of *Zasmidium* in China

**DOI:** 10.3897/BDJ.9.e59001

**Published:** 2021-01-07

**Authors:** Yuan-Yan An, Xiang-Yu Zeng, Kun Geng, Kevin David Hyde, Yong Wang

**Affiliations:** 1 Department of Plant Pathology, Agriculture College, Guizhou University, Guiyang, China Department of Plant Pathology, Agriculture College, Guizhou University Guiyang China; 2 Center of Excellence in Fungal Research, Mae Fah Luang University, Chiang Rai, Thailand Center of Excellence in Fungal Research, Mae Fah Luang University Chiang Rai Thailand; 3 School of Science, Mae Fah Luang University, Chiang Rai, Thailand School of Science, Mae Fah Luang University Chiang Rai Thailand; 4 Guiyang plant protection and inspection station, Guiyang, China Guiyang plant protection and inspection station Guiyang China

**Keywords:** one new species, asexual morph, Dothideomycetes, Mycosphaerellaceae, taxonomy

## Abstract

**Background:**

Two hyphomycetous species were collected from leaves of *Smilax china* (Liliales, Smilacaceae) and *Cremastra appendiculata* (Asparagales, Orchidaceae). ITS barcoding indicated that they belong to the genus *Zasmidium*.

**New information:**

Morphological data in combination with molecular phylogenetic analyses based on ITS, LSU and *rpb2* confirmed that our Chinese strains represented a new species, *Zasmidium liboense* and a new record of *Z. citri-griseum*.

## Introduction

The fungi of southern Asian are extremely diverse (Hyde et al. 2018, Cheek et al. 2020). During a survey of fungal diversity in ornamental plants in south-western China from 2017 to 2019, more than 2000 strains were obtained, which represented asexual morphs of both Ascomycota and Basidiomycota. Some new taxa were previously described by our research group as pathogens or endophytes (e.g. Liang et al. 2018, Long et al. 2019, Sun et al. 2020, Wijesinghe et al. 2020, Zhang et al. 2020).

The genus *Zasmidium* was established by Fries (1849) with *Z. cellare* (Pers.) Fr. as the type species. It is currently placed in the order Capnodiales within the Dothideomycetes (Hongsanan et al. 2020, Wijayawardene et al. 2020). Arzanlou et al. (2007) showed that *Zasmidium* was the oldest name for *Stenella*-like hyphomycetes within Mycosphaerellaceae, which are characterised by conidiogenous loci and conidia with truncate hila (Bensch et al. 2012). Hence, many former *Stenella* species were transferred to *Zasmidium* (Braun et al. 2010, Kamal 2010). Up to now, the number of accepted species in the genus is about 150 (Wijayawardene et al. 2020).

In this paper, we report on *Zasmidium* species found on medicinal plants in China. One new species (*Zasmidium liboense*) and one new Chinese record (*Z. citri-griseum*) are reported, based on evidence from morphology and molecular phylogeny.

## Materials and methods

### Samples collection and fungal strains isolation

The samples were collected in Xishuangbanna City, Yunnan Province, China. In order to obtain pure cultures, diseased leaf pieces of *Smilax china* (Liliales, Smilacaceae) and *Cremastra appendiculata* (Asparagales, Orchidaceae) were surface-disinfected following the method of Zhang et al. (2020). The strains were isolated using the single-spore method (Chomnunti et al. 2014). Colonies growing from single spores were transferred to potato-dextrose agar (PDA) and incubated at room temperature (28ºC). The holotype was deposited in the Herbarium of Department of Plant Pathology, Agricultural College, Guizhou University (HGUP). The ex-type cultures were deposited in the Culture Collection at the Department of Plant Pathology, Agriculture College, Guizhou University, P.R. China (GUCC) and the Mae Fah Luang University Culture Collection (MFLUCC) in Thailand.

### Morphological description

Morphological culture characters were recorded after 2–3 weeks of growth on PDA. Microscopic slides were prepared in lactophenol. Light microscopy observations were made using a BX53 compound microscopy (Olympus, Tokyo, Japan) at 1000× magnification. The morphology was observed using a compound microscope (OLYMPUS BX53) showing all necessary details of morphology and ontogeny of reproductive propagules. Measurements were made of 30 structures for conidia, hila and conidiophores. The new species name was submitted to MycoBank (www.mycobank.org).

### DNA extraction, PCR amplification and sequencing

Fungal cultures were grown on PDA at 28°C. When the whole Petri-dish (90 mm diam.) was nearly covered, fresh mycelia were scraped from the surface with sterilised scalpels. Genomic DNA was extracted using Fungus Genomic DNA Extraction Kit (Biomiga #GD2416, San Diego, California, USA) and following the manufacturer's instructions. PCR amplification of the internal transcribed spacer (ITS) region and the large subunit (LSU) of the ribosomal RNA gene was performed in a 25-μl reaction volume system as in Liang et al. (2018). Primers V9G and ITS4 (White et al. 1990, de Hoog and van den Ende 1998) were used to amplify the ITS and LSU1Fd and LR5 for the LSU (Vilgalys and Hester 1990, Crous et al. 2009). In addition, one protein-coding gene fragment, RNA polymerase II second largest subunit (*rpb2*), was amplified with the primers fRPB2-5F and fRPB2-7cR (Liu et al. 1999). Purification and sequencing of the PCR amplicons were undertaken by SinoGenoMax (Beijing, China). The resulting DNA sequences were submitted to NCBI GenBank (https://www.ncbi.nlm.nih.gov/genbank/) and their accession numbers are provided in Table 1.

### Phylogenetic analyses

Our newly-generated sequences were aligned by locus with ex-type and other representative sequences of *Zasmidium* species, which were downloaded from GenBank (Table 1). Alignments were made using the online version of MAFFT v. 7.307 (Katoh and Standley 2016) and manually improved, where necessary, using MEGA v. 6.06 (Tamura et al. 2013). Mesquite v. 2.75 (Maddison 2008) was used to concatenate the aligned sequences of the different loci. Ambiguous regions were excluded from analyses using AliView (Larsson 2014) and gaps were treated as missing data. DNA base differences of different gene loci between our strains and ex-type or representative strains of relative taxa are shown in Table 2. The alignment document is available in TreeBASE under the study ID 27250.

Maximum Parsimony (MP) analyses were performed in PAUP v. 4.0b10 (Swofford 2002), using the heuristic search option with 1,000 random sequence addition replicates and tree bisection-reconnection (TBR) as the branch swapping algorithm. Maxtrees was set at 10,000. The Tree Length (TL), Consistency Indices (CI), Retention Indices (RI), Rescaled Consistency Indices (RC) and Homoplasy Index (HI) were calculated for each tree generated.

Maximum Likelihood (ML) was inferred using the IQ-tree (Nguyen et al. 2015, Chernomor et al. 2016) under the Edge-linked partition model. Bootstrapping was done under 10000 ultrafast replicates (Hoang et al. 2018). ModelFinder (Kalyaanamoorthy et al. 2017) was used to select the best-fit partition model (Edge-linked) using BIC criterion. The best-fit model, according to BIC was: ITS: TNe+I+G4, LSU: TN+F+I+G4, *rpb2*: TN+F+I+G4.

For Bayesian Inference (BI), GTR+I+G was selected as the best model for all three loci (ITS, LSU and *rpb2*) as determined by MrModeltest v2 (Nylander 2004). BI analysis was undertaken using MrBayes v. 3.2.6 (Ronquist et al. 2012). Six Markov Chain Monte Carlo runs were launched with random starting trees for 1,000,000 generations and sampling every 1,000 generations. The first 25% resulting trees were discarded as burn-in.

## Taxon treatments

### Zasmidium liboense

Y.Y. An, Yong Wang bis & K.D. Hyde sp. nov.

D97D94FD-E802-51FF-B7FC-2455428769D8

#### Materials

- **Type status:** Holotype. **Occurrence:** catalogNumber: HGUP 1720.2; recordedBy: Wang Yong; **Taxon:** scientificName: Zasmidium liboense; kingdom: Fungi; class: Dothideomycetes; order: Capnodiales; family: Mycosphaerellaceae; genus: Zasmidium; **Location:** country: China; stateProvince: Guizhou; locality: Gold Thread Cave of Libo county; **Identification:** identifiedBy: Yuan-Yan An; dateIdentified: 2020; **Record Level:** type: ex-type cultures GUCC 1070.2; MFLUCC 20-0139; language: en

#### Description

**Figure 1.**
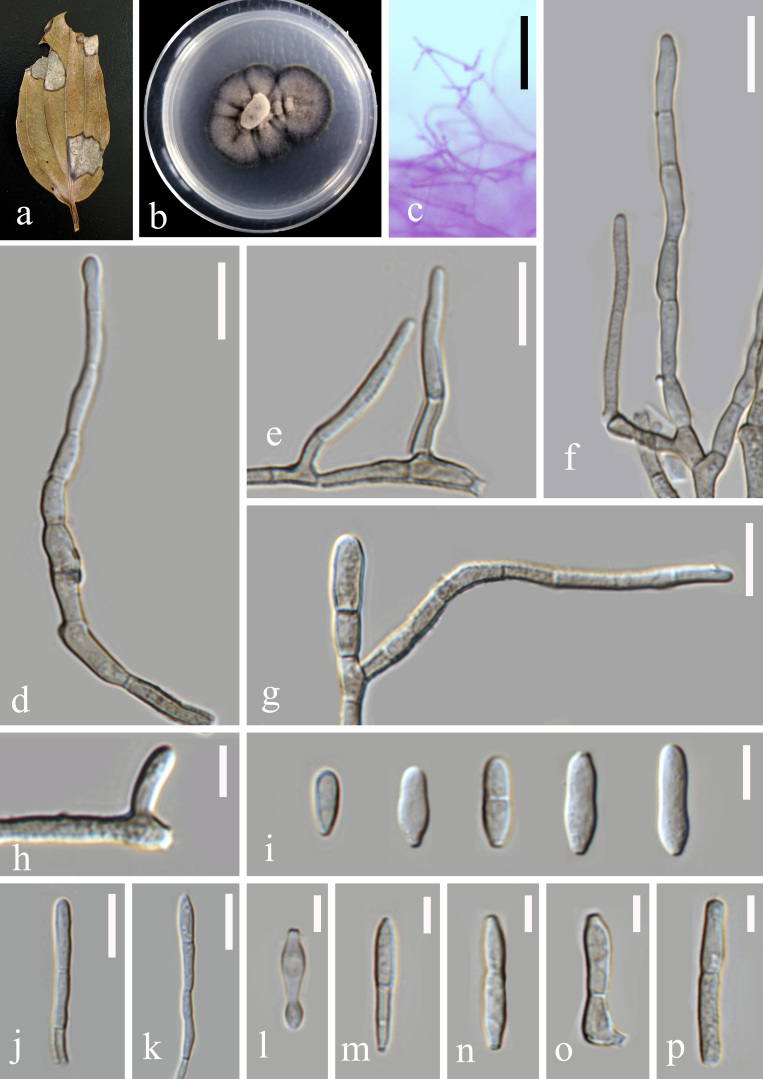
*Zasmidium liboense* (GUCC 1720.2). **a.** Leaf spot symptoms on the host; **b.** Culture on PDA; **c, d.** hyphae and conidiophores on PDA; **e–m.** Conidiophores and conidia; **n, o.** Conidiogenous cells and conidia; **p.** Conidia. Scale bars: c, d = 50 µm, e–j = 10 µm, k = 5 µm, l–p = 10 µm.

Pathogenic on the leaf spot of *Smilax china* (Liliales, Smilacaceae). Lesions on the upper leaf surface, scattered, distinct, irregular, rather large, the maximum length of the spot more than 20 mm, the edges of the disease reddish-brown, the centre dead to greyish-white, on the lower leaf surface similar. *Colonies* on PDA 10–15 mm diam. in 2 weeks, with an even, dark coffee margin. *Mycelium* composed of hyaline and pale brown to dark blackish-brown hyphae, verruculose, septate, branching, uniform in width, 2.5 μm. *Conidiophores* arising from hyphae, pale olivaceous brown to pale blackish-brown, finely verruculose, straight or slightly curved, dendritic rugged or rugose on the surface 20–350 × 1.5–3.5 μm. *Conidiogenous cells* integrated, apical, polyblastic, proliferating sympodially, with rim-like conidiogenous loci, thickened and darkened, located apically and lateraly as in a short rachis 1.5–2.5 μm diam. *Conidia* solitary, occasionally catenate, pale blackish-brown to pale olivaceous brown, verruculose, ellipsoidal, cylindrical to obclavate, base obconically truncate and apex rounded, straight or curved, 6–21 × 2–4 μm, 0–1-septate, sometimes constricted at septa, with hila thickened and darkened, 1–1.5 μm diam. (Fig. 1)

##### MycoBank Number

MB836278

#### Etymology

In reference to the location (Libo county, Guizhou Province), where the holotype was isolated.

### Zasmidium citri-griseum

(F.E. Fisher) U. Braun & Crous, IMA Fungus 5 (2): 337 (2014）

6940A98C-A2F7-5387-B8E6-56FC1C1650A1

#### Materials

- **Type status:** Other material. **Occurrence:** catalogNumber: HGUP 1507.3; recordedBy: An Yuan-Yan; occurrenceID: living culture GUCC 1507.3 and MFLUCC 20-0138; **Taxon:** scientificName: Zasmidium citri-griseum; kingdom: Fungi; class: Dothideomycetes; order: Capnodiales; family: Mycosphaerellaceae; genus: Zasmidium; **Location:** country: China; stateProvince: YunNan; locality: Xishuangbanna Dai Autonomous Prefecture; **Identification:** identifiedBy: Yuan-Yan An; dateIdentified: 2020; **Record Level:** language: en

#### Description

**Figure 2.**
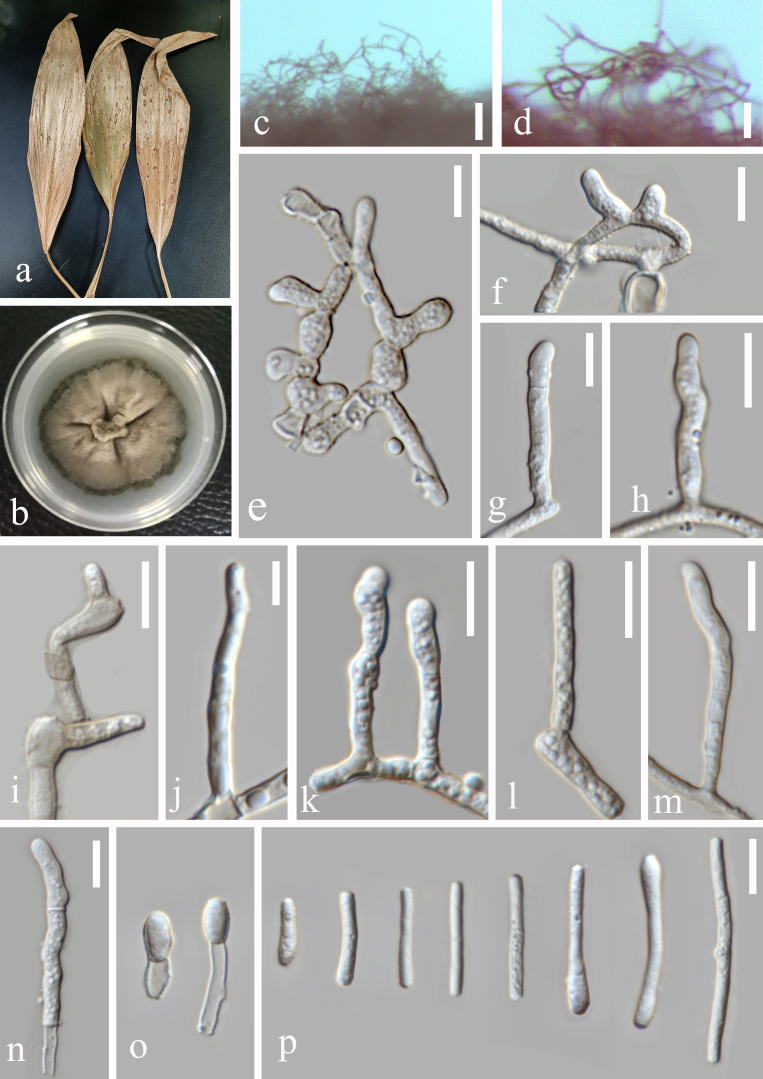
*Zasmidium citri-griseum* (GUCC 1507.3) **a.** Leaf spot symptoms on host; **b.** Culture on PDA; **c.** Hyphae and conidiophores on PDA culture; **d–h.** Conidiophores, conidiogenous cells and conidia; **i.** Conidia; **j, k.** Conidiogenous cells and conidia; **l–p.** Conidia. Scale bars: c = 100 µm, d–g =10 µm, h=5 µm, i, j = 10 µm, k–p = 5 µm.

Pathogenic on leaf spot of *Cremastra appendiculata* (Asparagales, Orchidaceae). Lesions on the upper leaf surface, scattered to confluent, distinct, angular, spots elliptic to suborbicular, reddish-brown to dark brown, 2–5 mm, on the lower leaf surface similar. *Colonies* on PDA 10–15 mm diam. in 2 weeks, with an even, dark-brown margin. Surface fold, with gully shape. *Conidiophores* arising singly as lateral branches of superficial hyphae, semi-macronematous to macronematous, mononematous, erect to flexuous, sometimes curved, unbranched, thick-walled, non-smooth surface, 1–3-septate, cylindrical, geniculate, brown, 35–135 × 5–7 µm. *Conidiogenous cells* integrated, terminal, 5–10 µm long, sympodial, polyblastic, cylindrical, geniculate, scars slightly thickened and darkened, 0.5–1.5 µm in diam. *Conidia* solitary in simple or occasionally branched chains, short to long cylindrical, some ends swollen straight to somewhat curved, 8–41 × 2–5 µm, unseptate, light brown, thin-walled, verruculose, 2–3.5 µm wide. (Fig. 2)

## Analysis

**Figure 3.**
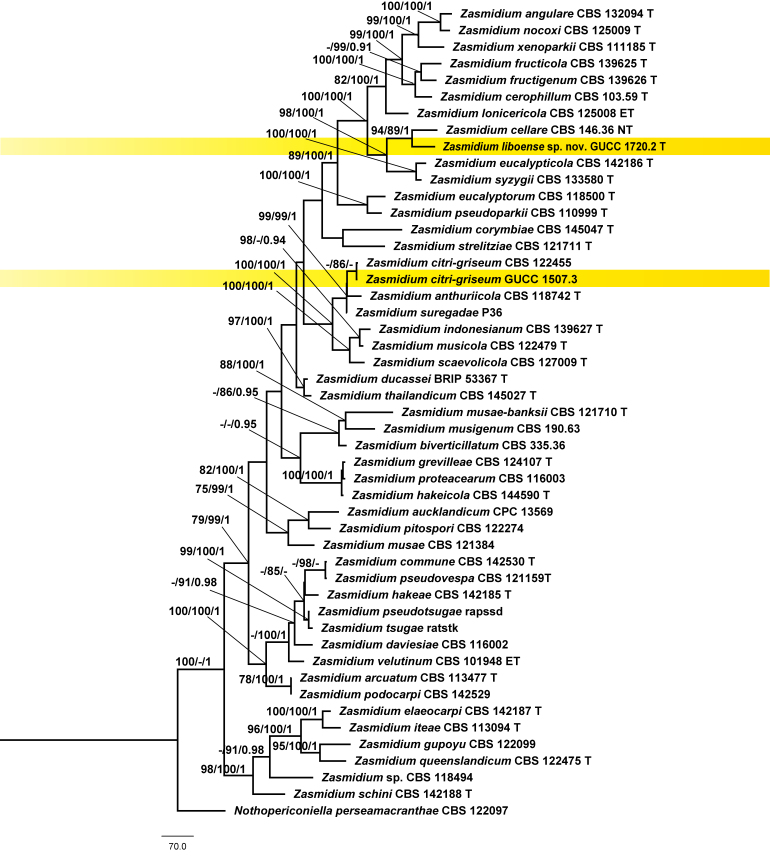
MP phylogeny of *Zasmidium* reconstructed from a three-locus dataset (ITS, LSU, *rpb2*). MP and ML bootstraps > 70 and BI posterior probabilities (PP) > 0.9 are placed close to topological nodes and separated by “/”. *Nothopericoniella perseamacranthae* (CBS 122097) was selected as outgroup.

Our final concatenated alignment included 2407 characters , viz. (ITS: 1–537, LSU: 538–1354, *rpb2*: 1355–2407), of which 645 were parsimony-informative characters. MP inference resulted in two equally-parsimonious trees (TL = 3705, CI = 0.35, RC = 0.59, HI = 0.20, RC = 0.65) and one of them was selected to show the topology (Fig. 3). Forty-eight *Zasmidium* strains clustered together as a clade, which received maximum support from both ML and BI analyses. *Zasmidium liboense* sp. nov. (GUCC 1720.2) was retrieved as sister taxon of *Z. cellare* (Pers.) Fr. (CBS 146.36) with high support (94 MP/89 ML/1.00 PP). Strain GUCC1507.3 grouped with a lineage consisting of *Z. anthuriicola* (U. Braun & C.F. Hill) Crous & U. Braun, *Z. citri-griseum* (F.E. Fisher) U. Braun & Crous and *Z. suregadae* Phengs., K.D. Hyde & U. Braun, with high support (99 MP/99 ML/1.00 PP). It was placed sister to *Z. citri-griseum* (CBS 122455) (86 ML).

A comparison of DNA bases (Table 2) demonstrated that, between *Z. liboense* (GUCC 1720.2) and *Z. cellara* (CBS 146.36), there were 23 bp differences in the ITS region, 19 in the LSU and 72 in the *rpb2*. The ITS sequence of GUCC 1507.3 differed in 8 bp from *Z. citri-griseum* (CBS 122455). Their LSU sequences were identical, whereas a single base pair difference was found in the *rpb2*.

## Discussion

Our phylogenetic analyses pointed out that *Zasmidium liboense* is different from *Z. cellare*. Morphologically, both species can be separated as well (Arzanlou et al. 2007). *Zasmidium liboense* produces conidia with 0-1 septa, whereas those of *Z. cellare* possess 0–1(–4) septa. Conidia of *Z. cellare* arise terminally or laterally and are subhyaline, whereas those of *Z. liboense* arise terminally and are pale blackish-brown to pale olivaceous brown. In addition, conidia of *Z. liboense* (6–21 × 2–4 μm) generally have larger dimensions compared to those of *Z. cellare* (6–9 × 1.8–2.5 µm). We also compared micro-morphology of our new taxon with 74 *Zasmidium* species, for which no sequence data are available. Most of those, 64 species, produce larger conidia with more septa compared to *Z. liboense*. In addition, *Z. araliae*, *Z. cerophilum* and *Z. litseae* all possess smaller conidia than *Z. liboense*. The conidia of *Z. capparacearum*, *Z. eriolobi*, *Z. gahniicola* and *Z. mitellae* have more septa compared to those of *Z. liboense*. *Zasmidium clusiae* produces conidia with basal unthickened hilum, whereas the conidia of *Z. liboense*, *Z. deightonianum* and *Z. oxycocci* have slightly or obviously thickened conidia. For these three species, the length of conidiophores can be used to distinguish amongst species: *Z. liboense* (20–350 μm), *Z. deightonianum* (10–30 μm) and *Z. oxycocci* (50–100 μm).

For strain GUCC 1507.3, our molecular phylogenetic data (Fig. 3, Table 2) showed that it was very close to *Z. anthuriicola*, *Z. citri-griseum* and *Z. suregadae*. *Rbp2* sequences of strain GUCC 1507.3 and *Z. citri-griseum* (CBS 122455) are identical, but different from *Z. anthuriicola* (CBS 118742). Currently, no *rpb2* sequences are available for *Z. suregadae*. Morphological comparison shows that conidia of our material (GUCC 1507.3) agree with the description of *Z. citri-griseum*; they are somewhat shorter although still in the range of measurements [*Z. citri-griseum*: 6–70(–120) × 2–4.5 μm] and they are aseptate [*Z. citri-griseum*: 0–1–(–6)-septate] (Braun et al. 2014). *Zasmidium citri-griseum* is one of the few species of *Zasmidium* with a wider host range. Braun et al. (2014) listed hosts in the Fabaceae, Musaceae and Rutaceae. The host of our Chinese material, *Cremastra appendiculata* (Orchidaceae), represents a new host family for this fungus.

## Supplementary Material

XML Treatment for Zasmidium liboense

XML Treatment for Zasmidium citri-griseum

## Figures and Tables

**Table 1.** Taxa used for molecular phylogenetic analyses and their GenBank accession numbers. (T) = ex-type strain.

Species name	Strain number	GenBank Accession numbers		
		LSU	ITS	*rpb2*
*Zasmidium angulare*	CBS 132094(T) = CPC 19042 = GA2 27B1a	JQ622096	JQ622088	MF951690
*Zasmidium anthuriicola*	CBS 118742(T)	FJ839662	FJ839626	MF951691
*Zasmidium arcuatum*	CBS 113477(T)	EU041836	EU041779	MF951692
*Zasmidium aucklandicum*	CPC 13569	MF951280	MF951409	MF951733
*Zasmidium biverticillatum*	CBS 335.36	EU041853	EU041796	–
*Zasmidium cellare*	CBS 146.36N(T) = ATCC 36951 = IFO4862 = IMI 044943 = LCP 52.402 = LSHBBB274 = MUCL 10089	EU041878	EU041821	MF951693
*Zasmidium cerophillum*	CBS 103.59(T) of *Acrotheca cerophila* = MUCL10034	GU214485	EU041798	MF951694
*Zasmidium citri-griseum*	CBS 122455 = CPC 15289 = X126	KF902151	KF901792	MF951695
	GUCC 1507.3	MT712179	MT683372	MT700485
*Zasmidium commune*	CBS 142530(T)	KY979820.1	NR_156003.1	–
*Zasmidium corymbiae*	CBS 145047(T)	NG_066279.1	NR_161118.1	MK047534.1
*Zasmidium daviesiae*	CBS 116002 = VPRI 31767	FJ839669	FJ839633	MF951698
*Zasmidium ducassei*	BRIP 53367(T)	–	NR_164517.1	–
*Zasmidium elaeocarpi*	CBS 142187(T) = CPC 16642	MF951263	MF951398	MF951699
*Zasmidium eucalypticola*	CBS 142186(T) = CPC 15149	MF951265	MF951400	MF951701
*Zasmidium eucalyptorum*	CBS 118500(T) = CPC 11174	MF951266	KF901652	MF951702
*Zasmidium fructicola*	CBS 139625(T) = CPC 24487 = ZJUM 80	KP895922	KP896052	MF951703
*Zasmidium fructigenum*	CBS 139626(T) = CPC 24471 = ZJUM 36	KP895926	KP896056	MF951704
*Zasmidium grevilleae*	CBS 124107(T) = CPC 14761	FJ839670	FJ839634	MF951705
*Zasmidium gupoyu*	CBS 122099 = RoKi 3022	MF951267	MF951401	MF951706
*Zasmidium hakeae*	CBS 142185(T) = CPC 15577	MF951268	MF951402	MF951707
*Zasmidium hakeicola*	CBS 144590(T)	NG_066335.1	NR_163384.1	MK442687.1
*Zasmidium indonesianum*	CBS 139627(T) = CPC 15300	KF902086	KF901739	MF951710
*Zasmidium iteae*	CBS 113094(T) = RoKi 1279	MF951271	MF951405	MF951711
*Zasmidium liboense* sp. nov.	GUCC 1720.2	MT712180	MT683373	MT700486
*Zasmidium lonicericola*	CBS 125008(T) of *Cladosporium lonicericola* = CPC11671	KF251787	KF251283	MF951712
*Zasmidium musae*	CBS 121384 = CIRAD 41 = X877	MF951272	EU514292	MF951713
*Zasmidium musae-banksii*	CBS 121710(T) = X1100	EU041852	EU041795	MF951716
*Zasmidium musicola*	CBS 122479(T) = X1019	MF951275	EU514294	MF951717
*Zasmidium musigenum*	CBS 190.63 = MUCL 9557	EU041857	EU041800	MF951718
*Zasmidium nocoxi*	CBS 125009(T) = CPC 14044	KF251788	KF251284	MF951719
*Zasmidium pitospori*	CBS 122274 = ICMP 17098	MF951276	MF951406	MF951720
*Zasmidium podocarpi*	CBS 142529	KY979821.1	NR_156004.1	–
*Zasmidium proteacearum*	CBS 116003 = VPRI 31812	FJ839671	FJ839635	MF951721
*Zasmidium pseudoparkii*	CBS 110999(T) = CPC 1087	JF700965	DQ303023	MF951723
*Zasmidium pseudotsugae*	rapssd	EF114704	EF114687	–
*Zasmidium pseudovespa*	CBS 121159(T) = AC0466	KF901836	MF951407	MF951724
*Zasmidium queenslandicum*	CBS 122475(T) = X1084	MF951277	EU514295	MF951725
*Zasmidium scaevolicola*	CBS 127009(T) = CPC 17344	KF251789	KF251285	MF951726
*Zasmidium schini*	CBS 142188(T) = CPC 19516	MF951278	MF951408	MF951727
*Zasmidium* sp.	CBS 118494 = CPC 11004	MF951279	DQ303039	MF951728
*Zasmidium strelitziae*	CBS 121711(T) = X1029	EU041860	EU041803	MF951729
*Zasmidium suregadae*	P36	KC677939.1	KC677914.1	–
*Zasmidium syzygii*	CBS 133580(T) = CPC 19792	KC005798	KC005777	MF951730
*Zasmidium thailandicum*	CBS 145027(T)	NG_066342.1	NR_164463.1	_
*Zasmidium tsugae*	ratstk	EF114705	EF114688	–
*Zasmidium velutinum*	CBS 101948(T) = CPC 2262	EU041838	EU041781	MF951731
*Zasmidium xenoparkii*	CBS 111185(T) = CPC 1300	JF700966	DQ303028	MF951732
*Nothopericoniella perseamacranthae*	CBS 122097 = RoKi 2995	GU452682	MF951354	MF951583

**Table 2.** The DNA base differences between our Chinese strains and related taxa in the three gene regions. Asterisks (*) denote our material.

**Species**	**Strain number**	**ITS (1-480 bp)**	**LSU** **(481-1254 bp)**	***RPB2*** **(1255-2355 bp)**
*Zasmidium citri-griseum* *	GUCC 1507.3	-	-	-
*Zasmidium citri-griseum*	CBS 146.36	8	1	0
*Zasmidium suregadae*	P36	6	0	/
*Zasmidium anthuriicola*	CBS 118742T	6	0	52
**Species**	**Strain number**	**ITS** **(1-475 bp)**	**LSU** **(476-1297 bp)**	***RPB2*** **(1298-2141 bp)**
*Zasmidium liboense* sp. nov.*	GUCC 1720.2	-	-	-
*Zasmidium cellare*	CBS 122455NT	23	19	72
